# Early monocular deprivation reduces the capacity for neural plasticity in the cat visual system

**DOI:** 10.1093/texcom/tgad017

**Published:** 2023-08-17

**Authors:** Jonathon Mark Henneberry, Joseph Elgallad, Seth Smith, Kevin R Duffy

**Affiliations:** Department of Psychology and Neuroscience, Dalhousie University, 1355 Oxford St., Halifax, NS B3H 4R2, Canada; Department of Psychology and Neuroscience, Dalhousie University, 1355 Oxford St., Halifax, NS B3H 4R2, Canada; Department of Psychology and Neuroscience, Dalhousie University, 1355 Oxford St., Halifax, NS B3H 4R2, Canada; Department of Psychology and Neuroscience, Dalhousie University, 1355 Oxford St., Halifax, NS B3H 4R2, Canada

**Keywords:** monocular deprivation, dLGN, visual cortex, neurofilament, visually-evoked potentials, neural plasticity, critical period

## Abstract

Obstruction of vision to one eye during early postnatal development elicits neural modifications in the visual system that can last a lifetime. Research in rodents has revealed that an early and transient monocular deprivation (MD) can produce an enduring alteration to the framework of neural connections within visual cortex. This lasting trace of early MD enables an enhanced effect of a second MD imposed on the same eye in adulthood. In the current study, we examined whether the modification of plasticity potential was bidirectional by assessing whether the effect of early and brief MD attenuated the impact of a subsequent MD when applied to the fellow eye. Results were clear in showing that animals with an early MD exhibited a smaller response to later visual deprivation of the fellow eye. Compared to controls, animals with a history of MD exhibited less atrophy of neurons, and a smaller loss of neurofilament labeling within the dorsal lateral geniculate nucleus. The shift in cortical ocular dominance elicited by MD was also smaller in animals with a prior MD. These results indicate that early MD elicits abiding and eye-specific neural modifications that can selectively alter plasticity potential in the visual system.

## Introduction

Disruption of clear and concordant binocular vision early in postnatal development sets into motion a cascade of neural changes that can impair visual performance for a lifetime. Monocular deprivation (MD) that occurs during the formative critical period of visual system development can shift cortical connections toward the non-deprived eye, leaving few neurons responsive to stimulation of the deprived eye (Wiesel and Hubel 1963b; Hubel et al. 1977; Dräger 1978; Mioche and Singer 1989). This imbalance in ocular dominance is precipitated by a weakening and loss of synaptic connections serving the deprived eye (Bear and Colman 1990), which is mirrored by a retrograde atrophy of deprived-eye neurons within the dorsal lateral geniculate nucleus (dLGN) of the thalamus (Wiesel and Hubel 1963b; Guillery and Stelzner 1970). The constellation of neural abnormalities provoked by MD can give rise to a severe and permanent impairment of visual function, amblyopia, that is most saliently characterized by reduced visual acuity in the deprived eye, and by loss of binocular vision (Dews and Wiesel 1970; Giffin and Mitchell 1978; Timney 1983; Vorobyov et al. 2007).

An early and brief MD imposed on young mice can have an impact on neural connections within visual cortex that lasts long after the deprivation is relieved (Hofer et al. 2009). The enduring neural modifications elicited by a transient MD can alter the response to a subsequent period of MD imposed much later in life (Hofer et al. 2006). Brief MD for 4–5 days in young mice can shift cortical ocular dominance so that neurons express a reduced response to stimulation of the deprived eye; however, simply opening the deprived eye completely restores normal responses. Imposing a second MD to the same eye in adulthood elicits a rapid and robust shift in ocular dominance that is not observed in adult mice without a history of early MD (Hofer et al. 2006). In other words, the first MD enhanced the effect of the second MD when it was applied to the same eye. Although it was shown that the enhanced effect of MD does not transfer to the fellow eye (Hofer et al. 2006), it is not known if plasticity was reduced with MD of the fellow eye because the effect of deprivation was assessed in adulthood when plasticity capacity is absent or negligible. In the current study we investigated whether early MD *reduced* plasticity capacity for the fellow eye. To that end we examined several markers of visual system plasticity in a highly visual animal, the cat, as a means of testing the hypothesis that an early and brief MD reduces the impact of a second MD when it is applied to the fellow eye.

Cats with ocular misalignment (strabismus) started early in development exhibit an attenuated effect of a subsequent MD on cortical ocular dominance compared to normal controls that receive the same MD (Mustari and Cynader 1981; Faulkner et al. 2005). This indicates that the experience of early eye misalignment reduces plasticity capacity and therefore weakens the impact of later visual deprivation. If the impact of fellow-eye MD is reduced in animals with a history of MD, this would raise the possibility that non-deprived eye neural circuitry is consolidated during the initial MD, and as a result acquired a resistance to the typical effects of MD when applied at a later age. This theoretical framework could help to explain results from classic studies of the critical period in monkeys and cats that suggest a shorter plasticity profile for the effects of reverse occlusion in comparison to MD alone (Blakemore and Van Sluyters 1974; le Vay et al. 1980; Olson and Freeman 1980).

The cat visual system has provided a reliable and robust means of assessing the impact of MD on neurons within the primary visual pathway (Wiesel and Hubel 1963a, 1963b; Giffin and Mitchell 1978; Rittenhouse et al. 1999), and has been a productive model of the human visual system. In this study, we assessed the capacity for visual deprivation to elicit neural changes in the visual system of cats that had a history of MD by measuring two anatomical hallmarks of deprivation: the reduction of neuron soma size and the loss of neurofilament protein within deprived-eye layers of the dLGN (Wiesel and Hubel 1963b; Guillery and Stelzner 1970; Bickford et al. 1998; Kutcher and Duffy 2007; Duffy et al. 2018). Visually-evoked potentials (VEPs) were also recorded from the primary visual cortex (V1) as a means of measuring the impact of MD on the balance of ocular dominance. Our findings indicate that an early MD can reduce the plasticity capacity of neural circuits serving the fellow eye.

## Methods

### Animals

Anatomical studies were conducted on 25 cats that were born and raised in a closed breeding colony at Dalhousie University. Some of the animals in this investigation were part of previous studies (Kutcher and Duffy 2007; O'Leary et al. 2012; Duffy et al. 2018; Duffy et al. 2023), and their tissues were reanalyzed alongside those reared specifically for the current study. All rearing and experimental procedures were conducted in accordance with protocols approved by the University Committee on Laboratory Animals at Dalhousie, and that conformed to guidelines from the Canadian Council on Animal Care. We compared the effect of 10 days of right-eye MD imposed at 8 weeks of age in two groups of animals that either received a left eye MD for 7 days at postnatal day 30 (Prior MD; n = 3) or did not receive a prior MD (No Prior MD; n = 4). In the second part of this study, we examined the influence of 10 days of right-eye retinal inactivation in animals that either had a prior left eye MD for 6 weeks starting at postnatal day 30 (Prior MD + Inactivation; n = 4) or did not (No Prior MD + Inactivation; n = 2). Our control groups consisted of normal animals (n = 7), MD for 7 days at postnatal day 30 (n = 3), and MD for 7 days at postnatal day 30 followed by binocular vision for 8 days (n = 2). The rearing histories of animals in this study are detailed in Table 1.

**Table 1:** Animal rearing conditions.

	Rearing Manipulations				
	Normal	1^st^ MD	BV	2^nd^ MD	Inactivation
**Normal**					
n = 2^a^	P0–P30				
n = 2^a^	P0–P70				
n = 3	P0–Adult				
**MD**					
n = 3^a^	P0–P30	P30–P37			
**MD + BV**					
n = 2^a^	P0–P30	P30–P37	P37–P45		
**Prior MD**					
n = 3	P0–P30	P30–P37	P37–P56	P57–P66	
**No Prior MD**					
n = 4	P0–P56	P56–P66			
**Prior MD + Inactivation**					
n = 4^a^	P0–P30	P30–P70	-	-	P70–P80
**No Prior MD + Inactivation**					
n = 2^a^	P0–P70	-	-	-	P70–P80

^a^Represents conditions for which tissue was acquired from our existing brain tissue bank.BV refers to binocular vision.

### Monocular deprivation

Animals were monocularly deprived under general gaseous anesthesia (3%–4% isoflurane in oxygen) and involved closure of the upper and lower palpebral conjunctivae with sterile 5–0 vicryl, followed by closure of the eyelids with 5–0 silk suture. Upon completion of the procedure, animals were administered oral Metacam (meloxicam; 0.05 mg/kg) for post-procedure analgesia, local anesthesia was produced with application of Alcaine sterile ophthalmic solution (1% proparacaine hydrochloride; CDMV, Canada), and infection was mitigated with a broad-spectrum topical antibiotic (1% chloromycetin; CDMV). Quality of the eye closure was monitored daily to ensure the lids were in good health and fully closed. Animals whose MD was followed by a period of binocular vision had their deprived eye opened under gaseous anesthesia (3%–4% isoflurane in oxygen) by simply removing the sutures. Alcaine eye drops were applied for local anesthesia, Metacam (0.05 mg/kg) administered for post-procedure analgesia, and a broad-spectrum topical antibiotic (1% chloromycetin) mitigated infection.

### Retinal inactivation

Animals subjected to monocular retinal inactivation were anesthetized with 3%–4% isoflurane and the right eye was given an intravitreal injection of TTX (ab120055; abcam, USA) solubilized in citrate buffer at 3 mM. Animals that received a prior MD had their deprived eye opened before the fellow eye was inactivated. For each animal, TTX dosage was scaled according to eye size linked with age (Thorn et al. 1976). We administered 0.5 μL of TTX per mm of vitreous chamber length. This dosage blocks action potentials of affected cells without obstructing critical cellular functions such as fast axoplasmic transport (Ochs and Hollingsworth 1971). Injections were administered through a small puncture made in the sclera located at the pars plana using a sterile 30-gage needle. Using a surgical microscope, the measured volume of TTX solution was dispensed into the vitreous chamber using a sterilized Hamilton syringe (Hamilton Company, USA) with a 30-gage needle (point style 4) that was positioned through the original puncture and about 5–10 mm into the chamber angled away from the lens. The total volume of TTX was dispensed slowly, and when complete the needle was held in place for about a minute before it was retracted. Following intraocular injection, topical antibiotic (1% chloromycetin) and a local anesthetic (1% proparacaine hydrochloride) were applied to the eye to prevent post-injection complications. Metacam (0.05 mg/kg) was provided for post-procedure analgesia. To achieve 10 days of complete inactivation, animals received five injections, one every 48 h, and for each injection the original puncture site was used to avoid having to make another hole. A single dose of TTX administered intravitreally eliminates visual responses for at least 48 h (Wong-Riley and Riley 1983; Stryker and Harris 1986; Linden et al. 2009; Fong et al. 2016). During the period of inactivation, we employed basic assessments of visual behavior to confirm inactivation while the non-injected eye was occluded with an opaque contact lens. We verified the absence of a pupillary light reflex as well as the lack of visuomotor behaviors such as visual placing, visual startle, and the ability to track a moving laser spot.

### Tissue preparation

In preparation for histology, animals were euthanized with a lethal dose of sodium pentobarbital (Pentobarbital Sodium; 150 mg/kg) and shortly after were exsanguinated by transcardial perfusion with approximately 150 mL of phosphate buffered saline (PBS) followed by an equal volume of PBS containing 4% dissolved paraformaldehyde. Brain tissue was immediately extracted and the thalamus was dissected from the remainder of the brain in order to prepare the dLGN for sectioning and histological processing. Tissue containing the dLGN was cryoprotected, then cut coronally into 25-μm thick sections using a sliding microtome. Sections for neurofilament labeling were cut at 50 μm. Tissue slices were stored at −20 °C immersed in an antigen preservative solution (Burke et al. 2009) until used for the study. Tissues examined in this study were all subjected to the same extraction and preparation procedures, the same storage settings, and sections from all animals were stained or labeled following the same protocol. Tissue sections collected from the left and right V1, as well as from the superior colliculus, were cryoprotected and stored for use in other studies.

### Histology

For each animal, 6 sections containing the left and right dLGN were mounted onto glass slides and stained with a 1% Nissl solution (ab246817; Abcam, USA). Stained sections were differentiated in 70% ethanol, then were dehydrated in a graded series of ethanols before clearing with Histo-Clear (National Diagnostics, Atlanta, GA). Sections were then coverslipped with Permount mounting medium (Fisher Scientific, Waltham, MA) and allowed to dry before microscopic evaluation.

Six sections containing the left and right dLGN from each animal were also selected for neurofilament labeling. Tissue slices were washed in PBS, then were placed free-floating in a PBS solution containing mouse monoclonal antibody (1:1,000) targeted against neurofilament protein (SMI-32, RRID:AB_509998; Biolegend, San Diego, CA) and left for 12 h. After being washed three times with PBS, sections were immersed for 1 h in a PBS solution containing biotinylated goat anti-mouse antibody (1:500) (115-065-003; Jackson ImmunoResearch, West Grove, PA). Sections were then rinsed with PBS and immersed in a PBS solution containing a mixture of avidin and peroxidase-conjugated biotin (PK6100; Vector Laboratories, Burlingame, CA) and left for 1 h. Immunolabeling was made visible by exposing tissue to a PBS solution containing hydrogen peroxide (1:1,000) and the chromogen, 3,3′-diaminobenzidine (0.5 mg/mL). Following a thorough wash with PBS, sections were mounted onto glass slides and allowed to dry, then were dehydrated in a graded series of ethanols before clearing with Histo-Clear (National Diagnostics, Atlanta, GA), and finally were coverslipped with Permount mounting medium.

### Quantification and analysis of anatomy

Measurements in this study were performed blind to each animal’s rearing condition. The cross-sectional area of neuron somata within A and A1 layers of the left and right dLGN was measured from 3–4 Nissl-stained sections using the nucleator probe available on a computerized stereology system (newCAST; VisioPharm, Denmark). All area measurements were performed using a BX-51 compound microscope with a 60× oil-immersion objective (Olympus; Markham, Ottawa, Canada). Neurons were distinguished from glial cells using established selection criteria (Wiesel and Hubel 1963a; Guillery and Stelzner 1970) that included measurement of cells with dark cytoplasmic and nucleolar staining, and with light nuclear staining (Duffy et al. 2012). Adherence to these criteria permitted avoidance of cell caps and ensured that measurements were taken from neurons cut through the somal midline. Approximately 1,000–1,500 neurons were measured from each animal. Assessments and measurements of the dLGN were made from sections that spanned coronal plane 6–7 (Sanderson 1971), which is positioned about midway along the anterior-posterior axis of the nucleus.

Sections labeled for neurofilament were imaged with an Olympus VS200 slide scanner using a 20× objective. Neurons labeled for neurofilament protein were counted using bioimaging analysis software, QuPath (Bankhead et al. 2017). Neurofilament-positive cell density was calculated for each layer separately by dividing cell counts by the area of each layer that was sampled. To avoid counting labeled cell caps, only neurons with distinct cytoplasmic labeling with weak or absent labeling in the nucleus were counted.

The effect of deprivation within each group was assessed using an unpaired Mann-Whitney test that compared measurements between eye-specific layers. Comparison of the effect of deprivation between groups was achieved with an ocular dominance index (ODI) calculated for each animal. For soma size and neurofilament data, the ODI calculation revealed the percentage difference between eye-specific dLGN layers for each animal separately:


\begin{align*} ODI&=\left(\left(\left( Non- Deprived\ Layer\ A+ Non- Deprived\ Layer\ A1\right)\right.\right.\\&-\left.\left( Deprived\ Layer\ A+ Deprived\ Layer\ A1\right)\right)/\\ &\left.\left( Non- Deprived\ Layer\ A+ Non- Deprived\ Layer\ A1\right)\right)\ast 100 \end{align*}

This metric enabled assessment of the effect of MD for each animal using measurements from the non-deprived eye as a reference. Our analysis strategy assists in mitigating the effect of having a low number of animals per group. Comparison of ODI across our prior and no prior MD groups was achieved using an unpaired Mann-Whitney test. Significance for all statistical tests was set at 0.05.

### Physiology

In preparation for measurement of VEPs, animals were induced with 3% isoflurane in oxygen that was reduced to between 1%–1.5% during recordings. Supplemental sedation was provided during recordings with intramuscular injection of acepromazine (0.06–0.1 mg/kg). Hair on the head was trimmed using an electric clipper, then a disposable razor was used to gently shave parts of the scalp where electrode recording sites were located. Two recording sites were positioned approximately 2–8 mm posterior and 1–4 mm lateral to either side of interaural zero over the presumptive location of right and left V1, and a third site over the midline of the frontal lobes was selected as a reference. Electrode sites were abraded with Nuprep EEG skin preparation gel (bio-medical, MI, USA), and were then further cleaned with alcohol pads. Reusable 10 mm gold cup Grass electrodes (FS-E5GH-48; bio-medical) were gently secured to each recording site using Ten20 EEG conductive paste (bio-medical, USA) that was applied to the scalp. Impedance of the recording electrode was measured in relation to the reference electrode to ensure values were below 5 kΩ. Electrophysiological signals were amplified and digitized with an Intan headstage (RHD2132; 20 kHz sampling frequency), then recorded using an Open Ephys acquisition board and GUI software (Open Ephys, USA). Visual stimuli were programmed in MatLab using the Psychophysics Toolbox extension (Brainard 1997; Pelli 1997), and presented on an LCD monitor (Dell 210-AMSR; 25″ display, 240 Hz refresh, 1,920 × 1,080 pixels) at a viewing distance of 70 cm. Steady state VEPs were elicited with full contrast square wave gratings with a 2 Hz contrast reversal frequency (Bonds 1984; Pang and Bonds 1991; Norcia et al. 2015). Gratings of different spatial frequencies (0.05, 0.1, 0.5, and 1 cycles/degree ) or a blank gray screen were presented in random order for 20 s each, with a blank gray screen also displayed during the 2 s interstimulus interval. Each stimulus was presented for at least 4 repetitions. The viewing eye was tested in isolation by placing a black occluder in front of the other eye during recording. The eyes were kept open with small specula, and were frequently lubricated with hydrating drops. Recording sessions lasted about 40 min and animal behavior was observed for at least an hour post-recording to ensure a complete recovery. The raw electroencephalogram was imported to MatLab where it was high-pass filtered above 1 Hz, then subjected to Fourier analysis (Bach and Meigen 1999; Norcia et al. 2015). The magnitude of VEPs was calculated as the sum of power at the stimulus fundamental frequency plus 6 additional harmonics (2, 4, 6, 8, 10, 12, and 14 Hz). Baseline nonvisual activity was calculated as the sum of power at frequencies just offset from the visual response (2.45, 4.45, 6.45, 8.45, 10.45, 12.45 and 14.45 Hz).

### Analysis of physiology

To assess possible differences in VEP power across the prior MD and no prior MD groups, an ODI (see above) was calculated to determine the percentage difference in VEP power between the left and right eye. The sum of VEP power measurements for all spatial frequencies presented to the non-dominant eye was subtracted from the sum of VEP power for the dominant eye, and this difference was divided by the sum of VEP power for the dominant eye. This produced a single number, calculated independently for each hemisphere, that was expressed as the percentage difference between the eyes. For this calculation, the dominant eye was defined as the one with the highest summed VEP power. Statistical comparison between groups was achieved by employing an unpaired Mann-Whitney test to determine if the shift in ocular dominance was significantly smaller for the prior MD group. VEP measurements from left and right V1 were treated as single observations, and statistical significance was set at 0.05.

## Results

### Effects of an early and brief MD are transient

**Fig. 1:**
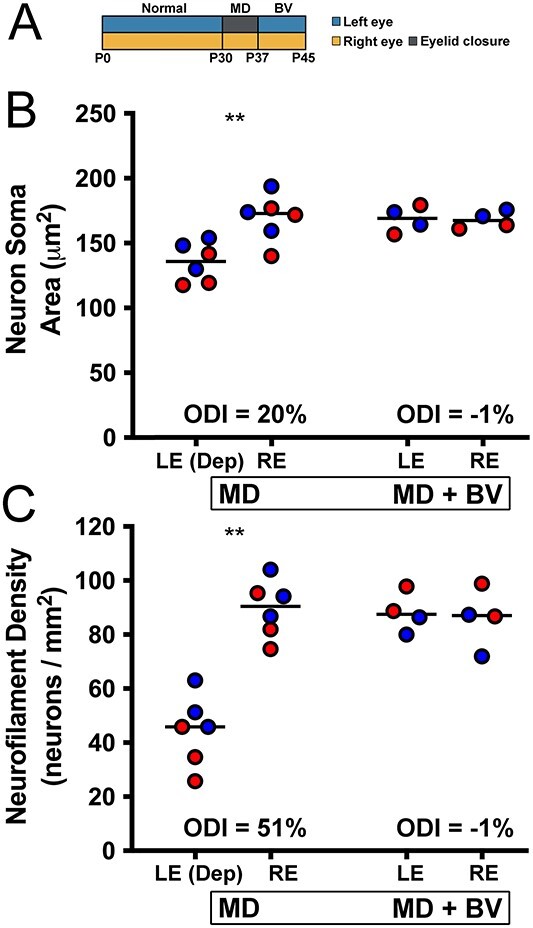
Anatomical effects of an early and brief MD resolve with provision of binocular vision. The experimental timeline for the MD condition and the MD with subsequent binocular vision (BV) condition is shown in A. Following 7 days of MD, the soma area of neurons in dLGN layers connected to the deprived eye was reduced by 20% when compared to neurons within layers serving the non-deprived eye (B). When the deprived eye was opened and binocular vision was provided for 8 days, deprived neurons fully recovered and matched the size of non-deprived counterparts (B). Similar results were obtained when the dLGN was examined for neurofilament protein. Following 7 days of MD there was a 51% reduction in the density of neurofilament-positive neurons within deprived layers of the dLGN (C). When 8 days of binocular vision was provided after the period of MD, there was a complete recovery of neurofilament density so that the deprived and non-deprived layers were indistinguishable. Red and blue data points indicate measurements from A and A1 dLGN layers, respectively. Double asterisks indicate statistical significance (p < 0.05).

To set the stage for our investigation of the influence of a prior MD on plasticity capacity, we needed to establish that the effect of a brief and early MD was transient. This was of paramount importance because it would indicate that animals given binocular vision after an early MD were appreciably normal according to our measurements before assessing the consequences of a second MD imposed later in development. A timeline for the MD-only and MD with binocular vision groups is depicted in Fig. 1A. Following 7 days of MD started at postnatal day 30, the soma area of neurons within deprived-eye layers of the dLGN was smaller compared to neurons within non-deprived layers (Fig. 1B). Stereological quantification of cross-sectional soma area revealed that deprived neurons (mean = 134 μm^2^; SD = 15 μm^2^) were 20% smaller than non-deprived neurons (mean = 169 μm^2^; SD = 18 μm^2^), and this difference was statistically significant (U = 6; p = 0.004). In a separate group of animals that received the same MD followed by 8 days of binocular vision, there was little difference between the size of dLGN neurons serving the two eyes. When quantified (Fig. 1B), neurons within previously deprived-eye layers (mean = 168 μm^2^; SD = 10 μm^2^) were comparable in size to those from non-deprived layers (mean = 167 μm^2^; SD = 7 μm^2^), and the layers were not significantly different (U = 7; p = 0.885). Therefore, consistent with previous reports (Blasdel and Pettigrew 1978; O'Leary et al. 2012; Duffy et al. 2015; Gotou et al. 2021), the reduction of soma size elicited by a short period of MD imposed early in postnatal development resolved with provision of binocular vision.

From the same animals described above, we assessed neurofilament labeling after 7 days of MD, as well as when 7 days of MD was followed by 8 days of binocular vision. A clear and substantial reduction in neurofilament labeling was observed within deprived-eye layers of the dLGN following 7 days of MD (Fig. 1C). Layers of the dLGN connected to the deprived-eye exhibited a 51% reduction of neurofilament-positive neurons (mean = 44 neurons/μm^2^; SD = 13 neurons/μm^2^) when compared to layers serving the non-deprived eye (mean = 90 neurons/μm^2^; SD = 11 neurons/μm^2^), which was a significant difference (U = 0; p < 0.001). In the group of animals that received 7 days of MD followed by binocular vision, there appeared to be a complete recovery from the loss of neurofilament (Fig. 1C). In these animals, the density of neurofilament-positive neurons was comparable between originally deprived dLGN layers (mean = 87 neurons/μm^2^; SD = 7 neurons/μm^2^) and layers serving the non-deprived eye (mean = 86 neurons/μm^2^; SD = 11 neurons/μm^2^), and this was not significantly different (U = 7; p = 0.885). Therefore, similar to measurements of soma size, the reduction of neurofilament labeling after early and brief MD recovered with provision of binocular vision.

### The impact of prior MD—soma size

**Fig. 2:**
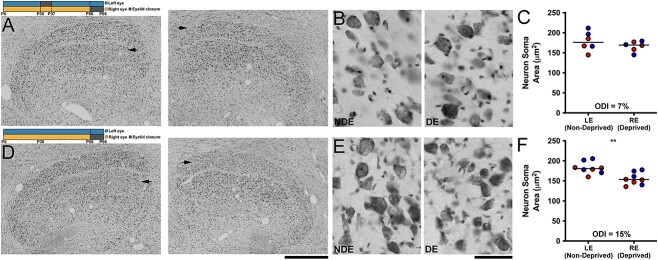
Prior MD reduces the impact that a later MD has on soma size in the dLGN. Animals in our first group were subjected to a 7-day MD of the left eye at 30 days of age, followed by an interval of binocular vision before a 10-day MD of the right eye at 8 weeks (timeline in A). This group showed only a slight alteration of Nissl staining within deprived-eye (DE) layers (arrows in A for the right and left dLGN) compared to non-deprived eye layers (NDE) that was just visible at low (A) and high (B) magnification. Stereological quantification of soma area reflected our qualitative observations by showing a small (7%) reduction of soma area in deprived-eye layers that was not statistically significant (C). A comparison group was identically reared except it did not receive an early period of MD (timeline in D). This group exhibited an obvious effect of MD that was evident at both low (D) and high (E) magnification, in which deprived-eye neurons (arrows in D) were clearly smaller than those located in layers serving the non-deprived eye. Quantification of soma area revealed that deprived neurons were 15% smaller than non-deprived neurons, and this was a significant difference (F). Scale bars = 1 mm (A and D) and 50 μm (B and E). Images in B and E were taken from non-deprived (left image) and deprived (right image) dLGN A layers. Red and blue data points indicate measurements from A and A1 dLGN layers, respectively. Double asterisks indicate statistical significance (p < 0.05).

We next examined whether an early and transient MD changed the efficacy of a later MD to modify soma size in the dLGN. The first group of animals examined were subjected to 7 days of MD at postnatal day 30, then were given binocular vision until 8 weeks of age before undergoing a second MD for 10 days, this time of the fellow eye. This group of animals exhibited a modest reduction of deprived-eye soma size that was evident upon microscopic inspection at both low and high magnification (Fig. 2A and B). Quantification of soma area (Fig. 2C) revealed a small modification in which deprived-eye neurons (mean = 166 μm^2^; SD = 13 μm^2^) were 7% smaller than neurons within dLGN layers serving the non-deprived eye (mean = 179 μm^2^; SD = 24 μm^2^). A Mann-Whitney test performed on data from the prior MD group revealed there was not a significant difference in soma size between deprived-eye and non-deprived-eye neurons (U = 14; p = 0.588). In contrast, the no prior MD group that was subjected to the same MD for 10 days also at 8 weeks of age, but that did not have a prior MD, revealed a more obvious alteration within the dLGN that was evident at low and high magnification (Fig. 2D and E). There was a clear reduction of soma area within deprived-eye layers. The soma area of deprived neurons (mean = 155 μm^2^; SD = 24 μm^2^) was on average 15% smaller than neurons serving the non-deprived eye (mean = 183 μm^2^; SD = 24 μm^2^). Whereas animals with a history of MD did not exhibit a significant alteration in soma size following a later MD, deprived neurons from animals without a prior MD were significantly smaller than those serving the non-deprived eye (U = 5; p = 0.003; Fig. 2F). These results indicated that the prior MD attenuated the impact on soma size of a second MD imposed later in development by about 50%.

### The impact of prior MD—neurofilament labeling

**Fig. 3:**
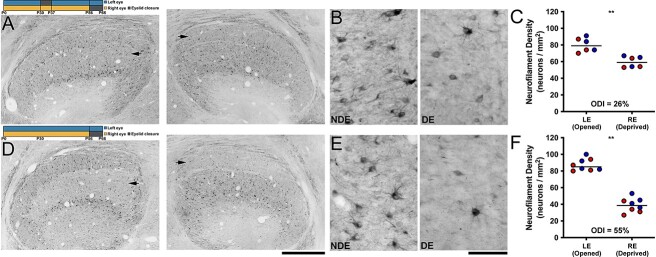
Prior MD reduces the impact that a later MD has on neurofilament labeling in the dLGN. Timelines and layout are identical to those described in Fig. 2. At low (A) and high (B) magnification, animals with an early history of MD showed a visible loss of neurofilament labeling within dLGN layers connected to the deprived eye (arrows in A for the right and left dLGN) compared to layers serving the non-deprived eye. Stereological quantification of soma area mirrored these qualitative observations by showing a 26% reduction of neurofilament positive density within deprived-eye layers that was statistically significant (C). The comparison MD group that did not have a history of early MD exhibited a more obvious loss of neurofilament labeling within deprived-eye layers (arrows in D), which was evident at low (D) and high (E) magnification. Quantification of neurofilament-positive cell density in this group revealed that deprived layers expressed a 55% loss relative to non-deprived layers and this was a statistically significant difference (F). The loss of neurofilament in this group was roughly double that measured from same aged animals that received an earlier MD. Scale bars = 1 mm (A and D) and 50 μm (B and E). Images in B and E were taken from non-deprived (left image) and deprived (right image) dLGN A layers. Red and blue data points indicate measurements from A and A1 dLGN layers, respectively. Double asterisks indicate statistical significance (p < 0.05).

Within the same group of animals that were examined for soma size modification, we assessed the influence of early MD on the efficacy of a later MD to modify labeling for neurofilament protein, a marker known to exhibit high sensitivity to MD (Bickford et al. 1998; Kutcher and Duffy 2007). In animals given a brief MD early in development, then a later MD of the fellow eye for 10 days at 8 weeks of age, we observed a loss of neurofilament within deprived layers that was detected upon viewing at low and high magnification (Fig. 3A and B). Quantification of neurofilament-positive cell density within layers of the dLGN (Fig. 3C) reflected our qualitative observations by revealing a 26% reduction within deprived-eye layers (mean = 59 neurons/μm^2^; SD = 7 neurons/μm^2^) compared to layers serving the non-deprived eye (mean = 80 neurons/μm^2^; SD = 8 neurons/μm^2^). Statistical comparison of neurofilament measurements revealed that neurofilament-positive cell density was significantly reduced in deprived layers compared to those serving the non-deprived eye (U = 0; p = 0.002). In the group of animals that received the same 10-day MD at 8 weeks of age but without a prior MD, the reduction of neurofilament labeling within deprived-eye dLGN layers was obvious, and was evident at both low and high magnification (Fig. 3D and E). The magnitude of the loss of neurofilament in this group appeared larger in comparison to the group that received a prior MD. Neurofilament-positive cell density within deprived-eye layers (mean = 39 neurons/μm^2^; SD = 9 neurons/μm^2^) was reduced by 55% relative to non-deprived layers (mean = 87 neurons/μm^2^; SD = 7 neurons/μm^2^), and this was also a statistically significant difference (U = 0; p < 0.001; Fig. 3F). As with our investigation of soma size, the reduction of neurofilament elicited by MD at 8 weeks of age was about half the size in animals that experienced a prior MD. That the prior MD group exhibited a significant loss of neurofilament but not a significant reduction in deprived-eye soma size indicates that modification of neurofilament labeling is a more sensitive marker for MD than alteration in soma size.

### Ocular dominance index: soma size and neurofilament labeling

**Fig. 4:**
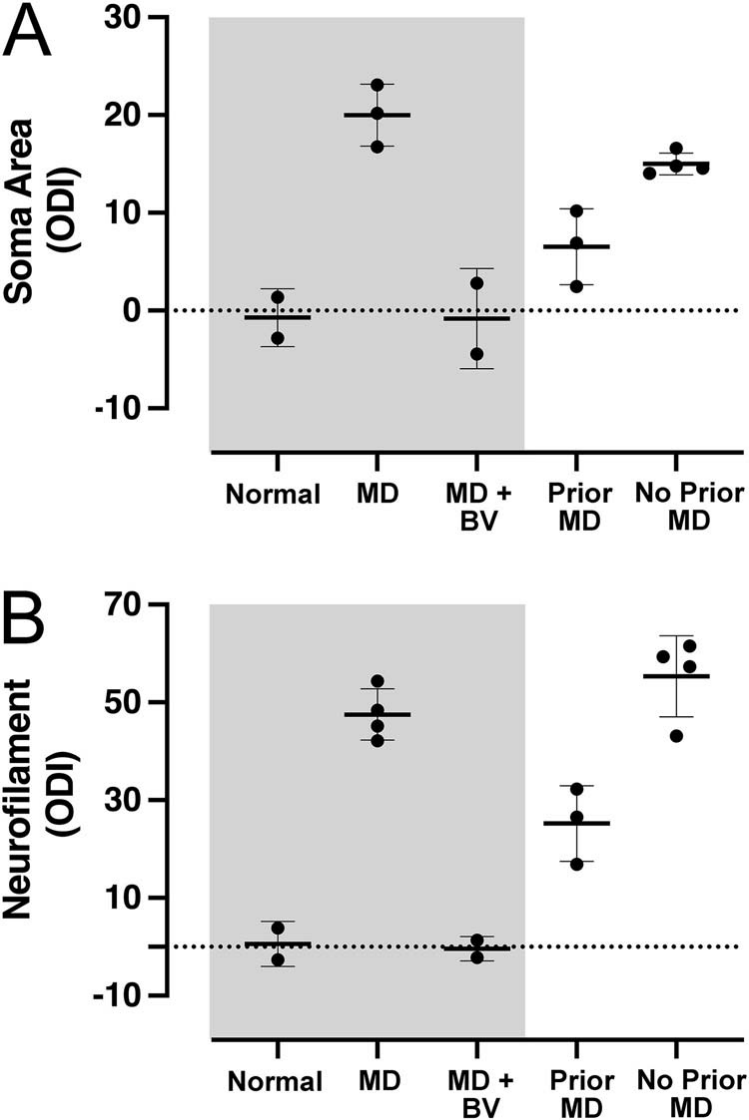
The effect of MD is reduced in animals with a history of MD. Control conditions (shaded area) demonstrate that the effect of an early and brief MD is erased with provision of binocular vision. An early period of MD reduces the size of neurons in deprived-eye layers of the dLGN, producing an imbalance of cell sizes between layers serving the right and left eye (A). This imbalance was restored to normal by opening the deprived eye and providing 8 days of binocular vision. A subsequent period of MD of the fellow eye elicited a small reduction of deprived cell size. Importantly, animals that were not subjected to a prior MD exhibited a reduction of deprived-eye cell size that was larger than the effect observed in animals with a prior MD. A similar pattern of results was observed when the same animals were examined for neurofilament labeling (B). Early MD reduced neurofilament-positive cell density within deprived layers of the dLGN by almost 50%, but this recovered and appeared normal within 8 days of providing binocular vision. Animals subjected to this early period of MD that at 8 weeks of age had their fellow eye deprived exhibited an effect on neurofilament labeling that was about half the size of the effect measured from age-matched animals that did not have a history of MD.

To compare the impact of MD on soma area and neurofilament labeling between the prior MD and no prior MD groups, we calculated the percentage difference between eye-specific dLGN layers for each animal (see ODI in Methods). We also calculated the percentage difference for animals in our control groups to summarize the effect of providing binocular vision after a brief MD early in postnatal development (shaded area in Fig. 4A and B). Whereas there was an even balance in soma size between eye-specific dLGN layers from normal animals, this was altered following 7 days of MD (Fig. 4A). Deprived neurons were reduced in size by about 20% compared to those serving the non-deprived eye. This asymmetry between eye-specific neurons was erased within 8 days of opening the deprived eye to provide binocular vision, which restored a normal balance between eye-specific layers. Animals that recovered from the initial MD, and then were subjected to a second MD of the fellow eye at 8 weeks of age (i.e. prior MD group) showed a smaller deprivation effect compared to the group of animals that did not receive a prior MD (Fig. 4A). Statistical comparison of these two groups with a Mann-Whitney test (one-tailed) revealed that the deprivation effect was smaller in the group of animals that received a prior MD (U = 0; p = 0.028). In the absence of a comparison group, the small effect observed in the prior MD group would likely have been attributed to the deprivation simply occurring at a later age; however, in aggregate these data demonstrate that the prior MD reduced the impact of the second MD.

The pattern of results for neurofilament labeling was similar to what was observed for soma size measurements (Fig. 4B). An early and brief MD reduced neurofilament-positive cell density within deprived-eye layers and altered the normal balance across eye-specific dLGN layers. Deprived layers had about half the number of immunolabeled neurons compared to non-deprived layers after MD; however, this imbalance was restored to normal within 8 days after the deprived eye was opened to provide binocular vision. The prior MD group that received a second MD at 8 weeks of age, this time of the fellow eye, exhibited a loss of neurofilament-labeled cells in deprived-eye layers. However, the size of this effect was considerably smaller than what was measured for the group that did not receive a prior MD (Fig. 4B). Statistical comparison of these two groups with a Mann-Whitney test (one-tailed) showed that the effect of deprivation was smaller in the group of animals that received a prior MD (U = 0; p = 0.028).

### The impact of prior MD—VEPs

**Fig. 5:**
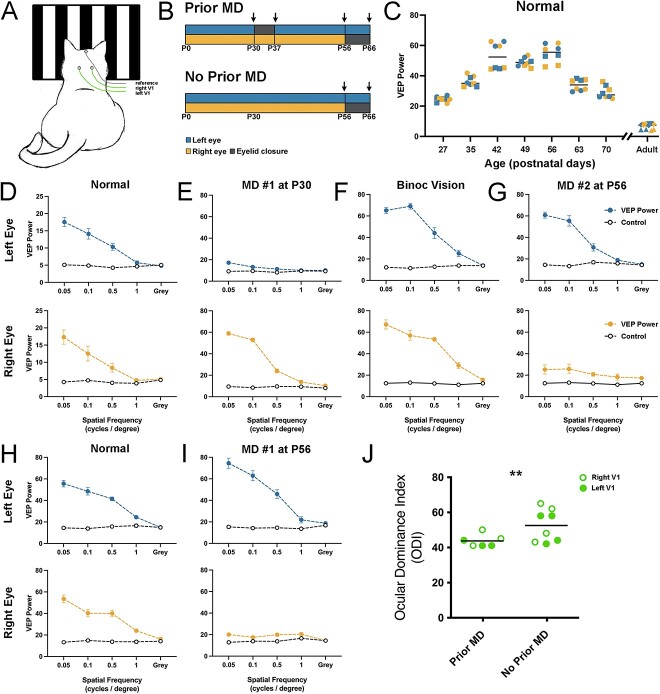
Early MD attenuates the shift in cortical ocular dominance elicited by a later MD of the fellow eye. Measurement of VEPs from right and left V1 were made using cup electrodes placed on the scalp while animals viewed grating stimuli of varying spatial frequency that were phased reversed at 2 Hz (A). The experimental timelines of Prior and No Prior MD groups are depicted in B, with arrows indicating the ages at which VEPs were measured. To provide a reference for our experimental data (C), VEP power data from five visually normal animals revealed a rise in power from P27 to P56 (n = 2), then a decline in VEP power to eventually reach very low levels in adulthood (n = 3). This decline in VEP power from its peak likely derives from a thickening of the skull with age, as well as a coincident growth of the temporal mastication muscle that is positioned under our recording site on the scalp. Measurement of VEP power elicited by separate stimulation of the left (blue trace) and right (yellow trace) eyes across 4 spatial frequencies and a control gray screen are presented with reference to non-visual baseline power (open circles) for an example animal in the Prior MD group (D). VEP power was comparable and balanced between the eyes before MD was imposed at P30, but after 7 days of left-eye MD there was a reduction in VEP power elicited by stimulation of the deprived eye (E). Following MD, provision of 3 weeks of binocular vision restored a normal level of VEP power to the originally deprived eye, which was in balance with the fellow eye (F). Imposition of a second MD, this time to the right eye for 10 days at 8 weeks of age, produced an attenuation of VEP power in the deprived eye that appeared less extreme at this older age than the effect of the first MD (G). Measurement of VEPs from the No Prior MD group were balanced between the right and left eyes before MD was imposed (H). After 10 days of MD started at 8 weeks of age, there was a large reduction of VEP power measured for the deprived eye, and the non-deprived eye showed an apparent potentiation relative to age-matched controls (I). To compare the extent of the shift in ocular dominance across groups (J), the summed VEP power from each eye was used to calculate an ocular dominance index for each animal, and for left (solid symbol) and right (open symbol) V1. The effect of 10 days of MD on ocular dominance was 20% smaller in the group of animals that received a prior MD (n = 3) compared to those that did not (n = 4). Different symbol shapes in C represent VEP measurements from different animals using the 0.05 spatial frequency. Duplicated pairs in C represent measurements from the right and left V1.

We investigated the impact of prior MD on the capacity for ocular dominance plasticity by measuring VEPs recorded from V1 (Fig. 5A). This was achieved by separate stimulation of the left and right eye in animals that were either subjected to 7 days of left eye MD at P30 or received no prior MD before receiving 10 days of right eye MD later in development. The timeline of procedures for both experimental groups is displayed in Fig. 5B, and the ages at which VEPs were measured is indicated with arrows. As a reference for comparison with our experimental animals, VEP recordings were made from V1 in a separate group of normal cats across various ages (Fig. 5C). At the youngest ages recorded in normal animals, VEP power was low, then increased to reach their highest level between P42 and P56. Thereafter, VEP power exhibited a progressive decline to reach very low levels in adulthood. Animals in the *Prior MD* group showed balanced VEP power between the left and right eyes before MD was imposed (Fig. 5D). Following MD for 7 days there was a shift in ocular dominance so that VEPs elicited by the deprived (left) eye were obviously attenuated, whereas VEPs serving the non-deprived eye appeared potentiated (Fig. 5E). In comparison to normal animals of similar age (Fig. 5C), non-deprived eye VEP power (measured at the 0.05 cycles per degree grating) was potentiated by about 75%. Provision of 3 weeks of binocular vision following an earlier episode of MD resulted in recovery of VEPs serving the originally deprived eye that were in balance with the fellow eye (Fig. 5F), and that were similar in power to those measured from normal controls of comparable age. Imposition of a second MD for 10 days at P56, this time of the right eye, produced an attenuation of VEP power elicited by stimulation of the deprived eye, whereas the non-deprived eye exhibited VEP power that appeared potentiated relative to similarly-aged normal controls (Fig. 5G). Animals in the *No Prior MD* group showed VEPs that were balanced between the eyes before MD was induced at P56 (Fig. 5H), and VEP power was in accordance with measurements from normal controls of about the same age. Following MD of the right eye for 10 days beginning at P56, VEPs elicited from the deprived eye were reduced in power, while VEPs elicited by the non-deprived eye were potentiated relative to similarly-aged normal controls by about double (Fig. 5I). We next compared the magnitude of the ocular dominance shift (ODI) produced by 10 days of MD imposed at P56 between our two MD groups (Fig. 5J). Animals that experienced a prior MD showed a 44% average difference between the eyes, whereas animals that had no prior MD exhibited a 53% difference between the eyes, which was only a modest divergence between the groups. The plasticity-attenuating effect of prior MD was clearly smaller when assessed with VEPs than with anatomy. Statistical comparison of the two groups to determine if animals with a prior MD showed a smaller effect revealed that the shift in ocular dominance was significantly smaller in animals subjected to a prior MD (Mann-Whitney test (one tailed); U = 9.5; p = 0.03). This effect was driven by two animals in the *No Prior*  *MD* group that exhibited large ODI values.

### Prior MD and retinal inactivation

**Fig. 6:**
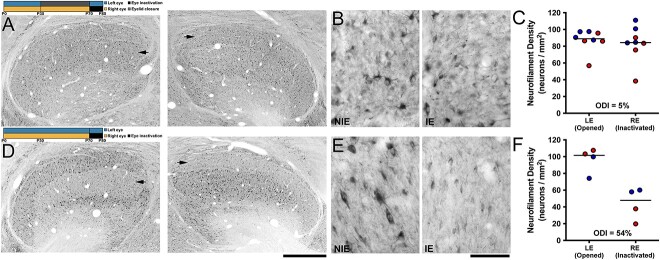
Early MD reduces the impact of a subsequent period of retinal inactivation on neurofilament labeling in the dLGN. Experimental timelines for each group are shown in A and D. Neurofilament labeling in the dLGN of an animal that received MD at postnatal day 30 for 6 weeks, then had their fellow eye inactivated for 10 days did not exhibit an obvious loss of immunolabeling in layers connected to the inactivated eye (arrows in A for the right and left dLGN). At higher magnification (B), roughly balanced labeling was observed throughout dLGN layers, and there was no obvious loss of neurofilament labeling within the layer serving the inactivated eye (IE) relative to the non-inactivated eye (NIE). This was confirmed by quantification of neurofilament-positive cell density, which showed a 5% difference between eye-specific layers that was not a significance difference (C). The group of animals that did not receive an early MD, but which did receive the same 10 days of monocular inactivation at the same age, expressed a clear loss of neurofilament labeling within layers serving the inactivated eye (arrows in D). This was obvious at high magnification where few neurofilament-positive neurons were observed in the inactivated-eye layers relative to layers serving the non-inactivated eye. Quantification of neurofilament-positive cell density revealed a 54% reduction within inactivated-eye layers (F), which was significantly different from fellow-eye layers. Scale bars = 1 mm (A and D) and 50 μm (B and E). Images in B and E were taken from dLGN A layers serving the non-inactivated eye (left image) and the inactivated eye (right image). Red and blue data points indicate measurements from A and A1 dLGN layers, respectively. Double asterisks indicate statistical significance (p < 0.05).

Results from the experiments described thus far indicated that a period of MD imposed early in postnatal development reduced the impact of a later MD to the fellow eye. To further test the effect of MD on plasticity potential, we assessed the impact of an identical period of monocular retinal inactivation in two groups of animals by measuring the consequence of inactivation on neurofilament labeling in the dLGN. The first group of cats received MD of the left eye for 6 weeks starting at postnatal day 30, then the deprived eye was opened and the fellow (right) eye was immediately inactivated for 10 days. The second group of animals received 10 days of right-eye inactivation at the same age, but this group did not receive an earlier MD. When 6 weeks of MD was followed by inactivation of the fellow eye for 10 days, neurofilament labeling in dLGN layers serving the inactivated eye (arrows in Fig. 6A) was not obviously reduced when examined at either low or high magnification (Fig. 6A and B), and there was a recovery of neurofilament labeling in dLGN layers connected to the closed eye as previously reported (Duffy et al. 2018). Quantification of neurofilament-positive cell density within layers serving the inactivated eye (mean = 83 neurons/μm^2^; SD = 21 neurons/μm^2^) and non-inactivated eye (mean = 87 neurons/μm^2^; SD = 13 neurons/μm^2^) revealed only a 5% reduction within inactivated-eye layers, and this appeared to produce a restoration of near-normal balance between the eye-specific layers. As can be seen in Fig. 6C, for unknown reasons one animal we examined expressed low neurofilament labeling in both A layers relative to other animals in the group (red symbols). The second group of animals that did not receive a prior MD before inactivation exhibited a substantial reduction of neurofilament labeling within dLGN layers connected to the inactivated eye (arrows in Fig. 6D), and this was particularly evident at high magnification (Fig. 6E). Quantification of neurofilament-positive cell density revealed a 54% reduction within dLGN layers connected to the inactivated eye (mean = 44 neurons/μm^2^; SD = 19 neurons/μm^2^) compared to those of the normal eye (mean = 96 neurons/μm^2^; SD = 15 neurons/μm^2^; Fig. 6F). A Mann-Whitney test (one-tailed) determined that the group with a prior MD expressed a significantly smaller effect in the dLGN compared to the group without a prior MD (U = 9.5; p = 0.03). Results from all of the groups examined in this study were consistent in showing that animals with a prior MD expressed a reduced capacity for plasticity of dLGN neurons serving the fellow eye.

## Discussion

In this study we investigated the hypothesis that abnormal visual experience early in postnatal life alters the capacity for neural modification and can lessen the impact of a subsequent episode of abnormal vision later in life. Results were clear in showing that animals subjected to a brief and transient MD initiated at the critical period peak exhibited an attenuated response to subsequent MD of the fellow eye when compared to a group of age-matched MD controls that had no history of MD. We next examined whether the plasticity-diminishing effect of early MD extended to a more extreme form of visual deprivation, namely, monocular retinal inactivation. Again, results were unambiguous in showing that the effect of monocular inactivation was reduced when it was preceded by a period of MD to the opposite eye. In aggregate, these results indicate that the impact of abnormal vision is informed by the history of visual experience that precedes it, and that early MD changes the visual system’s ability to change itself.

The reduced effect of MD and monocular inactivation that was precipitated by an earlier MD was observed when the final visual deprivation was imposed on the eye opposite to the one deprived first. This raises the possibility that early MD acts to consolidate neural connections serving the non-deprived eye, thereby conferring an acquired and enduring resistance to the effect of a subsequent episode of either MD or monocular inactivation. A lasting effect of early abnormal visual experience has been observed in juvenile barn owls subjected to a manipulation of the association between auditory cues and visual space using prismatic spectacles (Linkenhoker et al. 2005). This early abnormal visual experience elicited development of aberrant axonal projections to the external nucleus of the inferior colliculus that were presumed to accommodate the sensory discrepancy and facilitate adaptation. Importantly, these axonal projections persist long after the prisms are removed and normal visual behavior is restored (Linkenhoker et al. 2005). Exposure of these owls to the same abnormal sensory condition in adulthood results in readaptation (Knudsen 1998) that does not occur in normal adult owls (Brainard and Knudsen 1998). The readaptation is thought to be supported by a persistent neural framework that was acquired during initial prism wearing. In mice, early MD can also have a lasting impact on visual neural circuits in V1 (Hofer et al. 2009), and this can alter the visual system’s response to a subsequent episode of MD (Hofer et al. 2006). Brief MD (4–5 days) in young mice shifts ocular dominance away from the deprived eye, but simply opening the deprived eye restores normal ocular dominance (Hofer et al. 2006). Following recovery from the initial MD, mice that received a second MD to the *same* eye in adulthood exhibited a rapid and robust shift in ocular dominance that was not observed in adult mice without a history of early MD (Hofer et al. 2006). This effect appears partly rooted in an enduring alteration to dendritic spine density that results from the initial MD, providing a possible morphological substrate for the enhanced shift in cortical ocular dominance in mice with a history of MD (Hofer et al. 2009). The enhanced shift in ocular dominance observed in adult mice with a prior MD does not occur when the opposite (fellow) eye receives the later MD in adulthood (Hofer et al. 2006). However, it was not determined if MD of the fellow eye produced an attenuated effect because mice were examined in adulthood when plasticity capacity is negligible. Results from the current study indicate that the impact of early abnormal experience is bidirectional and eye specific. Our results extend those of Hofer et al. (2006) by revealing in a highly visual animal that neural circuitry serving the non-deprived eye is altered by early MD; it acquires a resistance to the effects of deprivation.

The plasticity-attenuating effect of a prior MD occurred after a second MD of the fellow eye, or after inactivation of the fellow eye with intravitreal administration of TTX. This suggests that disruption to normal vision during the critical period can reduce the impact of a subsequent episode of abnormal vision even if the two types of deprivation are different. Similarly, kittens rendered strabismic by myotomy during the critical period exhibited a smaller shift in cortical ocular dominance elicited by a subsequent MD (Mustari and Cynader 1981; Faulkner et al. 2005). Further, kittens with strabismus prior to MD showed a slower rate of recovery from the effects of MD, and recovery of VEPs elicited by the deprived eye was slower and less complete in animals with a prior strabismus. These effects were observed irrespective of the eye that received the MD, and were limited to the cortical hemisphere contralateral to the deprived eye (Faulkner et al. 2005). Notably, the effect of MD on cortical VEPs was not different in strabismic animals, which is at variance with the current study that showed a small but significant effect on VEPs. This may be due to the larger impact that early MD has on the visual system in comparison to strabismus (Mower and Duffy 1983), which could account for the smaller response to later MD in strabismic animals.

As has been suggested (Mustari and Cynader 1981; Faulkner et al. 2005), the protective effect that strabismus has on a later MD may derive from the breakdown of binocularity that occurs with strabismic rearing (Hubel and Wiesel 1965; Trachtenberg and Stryker 2001), which would preclude the competitive interactions between eye inputs that precipitate the typical effects of MD, and which are critical for recovery from MD (Kind et al. 2002). An assessment of this hypothesis can be extracted from a study by Blasdel and Pettigrew (1978) in which a single kitten was reared with alternating MD using an opaque occluder placed over each eye on successive days. This rearing procedure, if administered during the critical period, leads to near complete loss of effective binocular inputs within V1 (Hubel and Wiesel 1965; Blasdel and Pettigrew 1979). After rearing with alternating MD followed by 4 weeks of MD, the kitten showed a reduced physiological shift in cortical ocular dominance compared to age-matched MD-only controls (Blasdel and Pettigrew 1978). This suggests that a breakdown of binocularity induced by early MD could underlie the reduced effect of a later deprivation. If the effects of prior MD presented in the current study are the result of a loss of binocularity in V1, cats subjected to early MD that later receive another MD, but to the *same* eye, should also exhibit an attenuated response to the later MD because binocularity would be reduced equally for both eyes.

Our results were clear in showing that 1 week of early MD attenuated the effect of a subsequent episode of MD, but it did not completely block the effect of the second MD. The impact on soma size and neurofilament labeling were both reduced by about 50% in animals with prior MD. Animals subjected to monocular inactivation after MD showed a much larger plasticity reduction, with prior MD reducing the loss of neurofilament by about 90%. This was surprising because monocular inactivation can produce much larger anatomical effects in the dLGN compared to MD (Kuppermann and Kasamatsu 1983; Duffy et al., 2023). The near complete lack of neurofilament loss with monocular inactivation after a period of MD may derive from the longer duration of prior MD (6 weeks) that was imposed before inactivation was initiated. While the physiologic effects of MD can be observed within 2 days of MD onset (Mioche and Singer 1989), anatomical modifications are slower to manifest (Antonini and Stryker 1993; Duffy and Slusar 2009), with soma shrinkage and neurofilament loss progressing from 60%–80% effect magnitude to asymptotic levels between 7 and 10 days of deprivation onset (Kutcher and Duffy 2007). It is therefore conceivable that the extent of acquired resistance to a second MD of the fellow eye is governed by the duration of the MD that precedes it. This hypothesis is predicated on the assumption that the underlying neural adaptations elicited by the initial visual deprivation progress in accordance with the duration and possibly extent of visual occlusion.

The smaller loss of neurofilament in the dLGN of animals with a prior MD implicates neurofilament changes as a possible source for the reduced effect observed in this group. Neurofilament is a cytoskeletal protein enriched in axons and dendrites (Nixon and Logvinenko 1986; Millecamps et al. 2007), and assembles to form a stable and stationary intracellular scaffold that is well suited for maintaining the gross structural integrity of neurons (Morris and Lasek 1982; Yuan et al. 2009). Neurofilament levels in cat V1 accumulate postnatally following a profile inverse to that of the critical period (Song et al. 2015), and is reduced in cats exposed to complete darkness that exhibit enhanced plasticity potential (O'Leary et al. 2012; Duffy and Mitchell 2013). This implicates neurofilament as a plasticity inhibitor in the visual system (Duffy and Mitchell 2013; Hensch and Quinlan 2018). Neurofilament acquires enhanced stability through post-translational phosphorylation, a process that reduces its susceptibility to fragmentation by proteolysis (Pant 1988), and which is enhanced at ages beyond the critical period when plasticity potential is low (Liu et al. 1994; Song et al. 2015). The reduced loss of neurofilament observed within deprived layers of the dLGN from animals with prior MD may involve a precocious phosphorylation of neurofilament within non-deprived axons that is catalyzed by increased complexity of non-deprived axon terminal fields produced by the initial MD (Antonini and Stryker 1993). The reduced effect of MD on soma size in our prior MD animals may originate from a lasting stabilization of axons serving the originally non-deprived eye. The loss of neurofilament in deprived-eye dLGN layers may represent a destabilization of axons that could confer an enduring disposition for plasticity specific to neurons serving that eye.

The gold standard procedure for reversing the effects of MD is occlusion of the fellow eye (reverse occlusion). Despite this, emerging evidence in cats and mice suggests that reverse occlusion promotes a maladaptive plasticity phenotype (Balsor et al. 2019) that yields limited recovery from the physiological effects of MD (Kaneko and Stryker 2023; Martinez et al. 2023). That reverse occlusion exhibits weaker efficacy to elicit plasticity in comparison to MD is supported by results from the current study as well as from the long-standing observation that the critical period for susceptibility to reverse occlusion is shorter than that for MD (Blakemore and Van Sluyters 1974; le Vay et al. 1980; Olson and Freeman 1980). This could have implications for the treatment of human amblyopia because it suggests the abbreviated critical period of reverse occlusion may derive from the preceding MD itself. Consistent with this view is the finding that children with amblyopia that are treated unsuccessfully with patching of the fellow eye demonstrate less recovery with later patching compared to similarly-aged children with no history of patching (Scheiman et al. 2005; Scheiman et al. 2008; Holmes et al. 2011). Although it remains to be determined if these children express poor recovery simply because they are recalcitrant responders to patching, we suggest the possibility that the depth and duration of the initial amblyogenic event may alter plasticity potential and contribute to their poor recovery.

## CRediT authors statement

Jonathon Mark Henneberry (Conceptualization, Data curation, Writing—review & editing), Joseph Elgallad (Data curation, Writing—review & editing), Seth Smith (Data curation, Writing—review & editing), Kevin Duffy (Conceptualization, Formal analysis, Funding acquisition, Investigation, Methodology, Project administration, Resources, Supervision, Validation, Visualization, Writing—original draft, Writing—review & editing).

## Funding

This research was funded by grants from the Natural Sciences and Engineering Research Council of Canada (#RGPIN-2021-02798) and the Canadian Institutes of Health Research (#468904) to KRD.

*Conflict of interest statement:* The authors declare that the research conducted for this study has no commercial or financial relationships that could be viewed as a conflict of interest.

## Ethics statement

All procedures in this study were approved by the standing committee overseeing animal care and ethics at Dalhousie University, and they adhered to use guidelines directed by the Canadian Council on Animal Care.

## Data availability

All data presented in this manuscript are available upon request to KRD.
